# Integration of mRNA and miRNA analysis reveals the differentially regulatory network in two different *Camellia oleifera* cultivars under drought stress

**DOI:** 10.3389/fpls.2022.1001357

**Published:** 2022-09-30

**Authors:** Zhilong He, Caixia Liu, Zhen Zhang, Rui Wang, Yongzhong Chen

**Affiliations:** ^1^Research Institute of Oil Tea Camellia, Hunan Academy of Forestry, Changsha, China; ^2^National Engineering Research Center for Oil Tea Camellia, Changsha, China

**Keywords:** *Camellia oleifera*, miRNAs, regulatory network, drought stress, photosynthesis

## Abstract

Camellia oleifera Abel. (*C. oleifera*) is an edible oil tree species that provide an important guarantee for targeted poverty alleviation strategy in China. Severe difficulties in irrigation leading to drought stress have become a major obstacle to the development of the *C. oleifera* planting industry. Breeding of drought-tolerant cultivars is the main idea for solving the problem of water shortage stress in *C. oleifera* cultivation. The photosynthetic physiology traits of *C. oleifera* cultivars ‘Xianglin No.1’ and ‘Hengdong No.2’ were affected by drought stress to different degrees, which demonstrated that the two cultivars suffered different degrees of damage. In the present study, we applied mRNA-seq and miRNA-seq to analyze the difference in molecular responses between drought stress and control, drought-tolerant and -sensitive cultivars, at mRNA and miRNA levels. The differentially expressed genes (DEGs) involved in photosynthesis-related, porphyrin, and chlorophyll metabolism, circadian rhythm system, and plant hormone signal transduction pathways were identified that might be candidates for drought stress tolerance genes. Subsequently, the miRNA-mRNA regulatory networks connected the differentially expressed miRNAs (DEMs) to their predicted target genes were established. miR398 and miR408-3p in *C. oleifera* showed that associated with the response to drought stress by negatively regulating genes encoding Downy Mildew Resistance 6 (DMR6) and Enhanced Disease Resistance 2 (EDR2), respectively, which might further improve drought tolerance *via* crosstalk between different stress-responsive pathways. The quantitation results of miRNA and mRNA were validated by quantitative real-time PCR (qRT-PCR). In summary, the integrated mRNA-seq and miRNA-seq analysis deepen our understanding of the regulatory network response to drought stress and variety-specific responses improving drought tolerance in *C. oleifera*.

## Introduction

*Camellia oleifera* Abel. (*C. oleifera*) is an edible oil tree species with growing commercial, medic, cosmetic and ornamental values in recent years that provide an important guarantee for targeted poverty alleviation strategy in China (He et al., 2020). It has been widely cultivated in south-central and southern China, such as Hunan, Jiangxi, Guangxi, Zhejiang, Fujian, and Hainan provinces (Liu et al., 2018). The area of which *C. oleifera* plantation approximately reached 4.4 million hectares with an annual output of over 2.6 million tons of seeds consequently oil yields more than 0.65 million tons (Chen et al., 2020).

Drought stress is one of the most crucial environmental factors impairing photosynthesis, thereby limiting plant growth and yield (Muhammad et al., 2020). Accordingly, drought stress is regarded as one of the main threats to food security in the prevailing climate change era (Siddique et al., 2016). Plants have developed strategies to cope with drought stress, to ensure survival under severe drought stress, which includes stomata closure, osmotic adjustment, and enhanced tolerance level (Muhammad et al., 2020). Benefiting from recent advances in high-throughput sequencing, research to identify drought-related genes and miRNA regulatory- networks to understand various drought response mechanisms has made great progress in plants, such as cowpea (Mishra et al., 2021), durum wheat (Liu et al., 2020), and tobacco (Chen et al., 2017). MicroRNAs (miRNAs) are a class of small RNAs that are increasingly being recognized as important modulators of gene expression at the post-transcriptional level. Many miRNAs are involved in drought stress responses, including ABA response, auxin signaling, osmoprotectant, and antioxidant defense, by downregulating the respective target genes encoding regulatory and functional proteins (Ding et al., 2013).

Although *C. oleifera* has been classified as a drought-tolerant tree species, it is mainly planted on hills and mountains distributed in south China, which has severe difficulties in irrigation. In addition, the southern monsoon climate region has high temperatures and little rainfall in summer, resulting in drought stress becoming a major obstacle to the development of the *C. oleifera* planting industry.

This study aimed to identify the main candidate genes and miRNAs associated with the different responses between two genotypes of *C. oleifera* cultivars when they suffered from severe drought stress and to preliminarily reveal the potential molecular mechanisms of miRNA-mRNA regulatory networks that participated in the formation of drought tolerance.

## Materials and methods

### Plant material and treatments

Two Oil-tea cultivars, namely, *C. oleifera* cv. ‘Xianglin No. 1’ (XL1) and ‘Hengdong No. 2’ (HD2) were used in this study. The two cultivars were selected by applying a previous study in which twelve *C. oleifera* cultivars were subjected to severe artificial drought stress until death due to water cutoff. The growth status of the seedlings was observed every 3 days to evaluate the degree of drought stress. After 18 days of continuous water withholding treatment, the seedling survival rate of XL1 was about 80%, while it was zero for HD2, which showed the most obvious difference in survival rate, reflecting that XL1 was more tolerant to drought stress than HD2. The artificial simulated drought stress was conducted by withholding water to an extreme soil water content of 25 percent of the field capacity for 12 days with 3-year-old seedlings, while the control group of seedlings was irrigated daily to normal field capacity, in the mid to late July 2021 in the glasshouse at Hunan Academy of Forestry Sciences which geographical coordinates are 113°01′ east longitude and 28°06′ north latitude. Leaves of two cultivars at 0, 4, 8, and 12 days after drought stress were used for photosynthetic physiological traits measurement and high throughput sequencing library construction. Each cultivar in this experiment has three biological replicates.

### Measurement of the photosynthetic physiological traits

The gas exchange indicators include the net photosynthetic rate (P_n_, μmol^⋅^m^–2^s^–1^), stomatal conductance (G_s_, mmol^⋅^m^–2^s^–1^), intercellular CO_2_ concentration (C_i_, μmol^⋅^mol^–1^), and net transpiration rate (T_r_, mmol^⋅^m^–2^s^–1^) were measured on the morning during drought stress treatment by using a gas-exchange system LI-6400XT (LI-COR, Lincoln, NE, USA) with the parameter settings were 1,000 μmol photons m^–2⋅^s^–1^ and 400 CO_2_ μmol^⋅^mol^–1^ (He et al., 2021). Dark-adapted maximum photochemical efficiency of PSII (Fv/Fm) was measured with shaded leaves of each seedling.

### RNA libraries construction and sequencing

Twenty-four leaf samples collected from seedlings of the two *C. oleifera* cultivars that suffered drought stress with three biological replicates were used for RNA library construction and deep sequencing analysis. The mRNA and miRNA library preparations were analyzed on the Illumina Hiseq 2500 platform (Illumina, San Diego, CA, USA) at Allwegene (Beijing, China) with pair-end (2 × 150 bp) and single-end (50 bp) strategies, respectively. Clean data from sequencing were obtained by conducting a series of standardized processing of the raw data, then a certain length range from clean reads was chosen to do all the downstream analysis.

### Target prediction and functional annotation

The clean reads obtained from miRNA-seq were mapped to the reference sequence by Bowtie (Langmead et al., 2009) without mismatch to analyze their expression and distribution on the reference. miRBase20.0 was used as a reference to obtain the potential miRNA with modified software mirdeep2 and srna-tools-cli (Friedlander et al., 2012). The available software miREvo (Wen et al., 2012) and mirdeep2 (Friedlander et al., 2012) were integrated to predict novel miRNA. Predicting the target gene of miRNA was performed by psRobot (Wu et al., 2012) for plants. All these analyses were run automatically with default parameters.

Gene Ontology (GO) (Young et al., 2010) and KEGG (Kanehisa et al., 2008) pathway enrichment analysis were conducted on the expressed genes identified from mRNA-seq and target gene candidates of expressed miRNAs identified from miRNA-seq.

### Differentially expressed genes and microRNAs analysis

Clean data from mRNA-seq were aligned to the reference genome (*Camellia sinensis* genome GCF_004153795.1) by TopHat (v2.1.0). HTSeq was used to calculate the number of clean reads aligned to the characterized gene loci, and DESeq2 was then applied to identify the differentially expressed genes (DEGs) with cutoff | log_2_ fold change| ≥ 2 and *p*-value ≤ 0.05. To identify drought-responsive miRNAs, we selected miRNAs with TPM > 1 in at least one of the sample pairs to be analyzed. The criteria to consider a miRNA to be drought-responsive are | log_2_ fold change| ≥ 1 and *p*-value ≤ 0.05.

### Co-expression network analysis for construction of modules

We conducted the weighted gene co-expression network analysis (WGCNA) with R package (v1.71) (Langfelder and Horvath, 2008). A total of 2,550 genes that differentially expressed among XL1 and HD2 during different time points of artificial simulation drought stress were selected for further analysis. The gene expression values were imported into WGCNA to construct co-expression modules using the automatic network construction function blockwise modules with default settings, except that the power is 6, TOM Type is unsigned, mergeCutHeight is 0.60, and minModuleSize is 50. The expression profile of merged modules was summarized by the first principal component (module eigengenes, MEs), and the MEs of merged modules were correlated with the physiological traits to find the key modules associated with drought adaptation potential in *C. oleifera*. The top ten genes with maximum intra-modular connectivity were considered hub genes (Wisniewski et al., 2013) and visualized by Cytoscape (version 3.9.2) (Shannon et al., 2003).

### Real-time quantitative RT-PCR

The gene expression levels of four differentially expressed miRNAs including miR408, miR166, and miR398, together with their predicted target genes that were identified from the differentially expressed genes under drought stress among XL1 and HD2, as well as photosynthesis-related genes, plant hormone signal genes, and circadian clock genes that respond to drought stress were determined by qRT-PCR, within use the leaf samples that were the same as those applied to high throughput sequencing according to the method previously reported (He et al., 2021). The primers used for qRT-PCR were determined on the 5′ region of the candidate genes with putative amplicons of 150–250 bp and listed in Table 1.

**TABLE 1:** Information on primers used in the quantitative real-time PCR (qRT-PCR) analysis.

Primer Name	Sense primer	Anti-sense primer	Gene id
qLHY	CTCGTCTGCTACTGCTTCTCTG	CCTCTCCGACTATCCACAATGC	LOC114289998
qTOC1	ATGTGGAGGAGAAGGCGAATG	CAGGAGCAGCAGCAGTGG	LOC114285600
qRbcS	CTCGTCGCAGGTGTTGAAGG	CTGAATGATGAGTCACAATGAGTCC	LOC114317534
qFBA	TGAGAACACCGAGCACAACC	CGAAGAGAATGACACCAGAGAGG	LOC114315679
qPYL6	GAAGGGAACACTACAGACGAGAC	CGAGCCACGAGCAGGATTG	LOC114301961
qDELLA	GGAGGAGGAGGCGGAATGG	CAGCAGCGAGTGGTGAAGC	LOC114311179
qDMR6	TCAAGGATGGCAAGTGGATGG	GGCAGAGGAAAGAGGCTATGG	LOC114259217
qEDR2	AACCAGAATGCCAACAATCAAGC	TCCATCTACCTCCTCCACTAAGC	LOC114262251
qGene922	CAGAGTGTAGAGTCAAGGAGGAG	GGAGGATGAATTGGAAGATGAAAGC	LOC114285922
qGene616	TTAGCGGTTCCAGCGACTTC	CATTATCAGCATCAGCCAGATTAGC	LOC114322616
qmiR398	CGCGTGTGTTCTCAGGTCG	AGTGCAGGGTCCGAGGTATT	vvi-miR398b
qmiR408	GCGTGCACTGCCTCTTCC	AGTGCAGGGTCCGAGGTATT	stu-miR408b
qmiR166	GCGTCTCGGACCAGGCTT	AGTGCAGGGTCCGAGGTATT	aof-miR166b
qNovel146	CGCGTTCCTGTTGTGCCTG	AGTGCAGGGTCCGAGGTATT	novel_146

### Data analysis

SPSS was used for statistical analyses of the data of the CO_2_ exchange, and the analysis of variance (ANOVA) was conducted by using Duncan’s multiple range test, different lowercase letters indicate significant differences at *P* < 0.05.

## Results

### Verification of drought tolerance of the two *Camellia oleifera* cultivars

**FIGURE 1:**
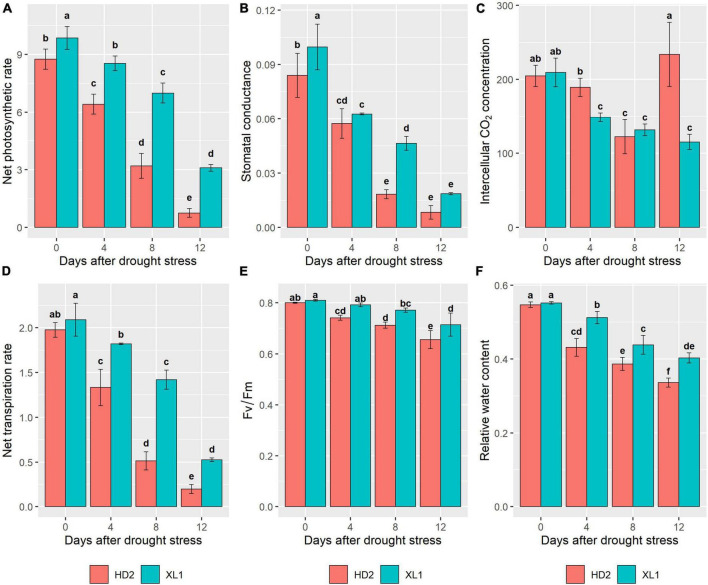
The differences in photosynthetic parameters between XL1 and HD2 during the drought stress treatment. Net photosynthetic rate (P_n_), Stomatal conductance (G_s_), Intercellular CO_2_ concentration (C_i_), and Net transpiration rate (T_r_) of both XL1 and HD2 leaves were measured by LI-6400XT between 8 and 11 a.m. local time. **(A)** Mean of the P_n_, **(B)** mean of the G_s_, **(C)** mean of the C_i_, **(D)** mean of the T_r_, **(E)** mean of the Fv/Fm, and **(F)** mean of the relative water content. Each data point represents the average of three biological replicates. Error bars represent mean ± SE. Different lowercase letters indicate significant differences at *P* < 0.05.

To verify the different physiological responses to drought stress between the two cultivars, photosynthetic traits were measured in this study. The results showed that drought stress induced a significant reduction of P_n_, G_s_, C_i_, and T_r_ in both two cultivars (Figure 1). In the comparison of the two cultivars, XL1 kept higher levels of Fv/Fm after 4, 8, and 12 days of drought stress treatment (0.79, 0.77, and 0.71) than those of HD2 (0.74, 0.71, and 0.66), respectively. The leaf relative water content of XL1 after 4, 8, and 12 days of drought stress treatment (0.51, 0.44, and 0.40) also maintained higher levels compared to those of HD2 (0.43, 0.39, and 0.34), respectively.

### Transcriptomic changes responded to drought stress in *Camellia oleifera*

**FIGURE 2:**
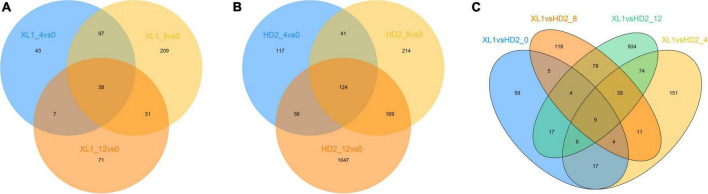
The venn diagram of differentially expressed genes (DEGs). **(A)** DEGs response to drought stress in XL1, **(B)** DEGs response to drought stress in HD2, **(C)** DEGs between XL1 and HD2 during drought stress.

High-throughput Illumina sequencing yielded a total of 883,425,114 clean reads with Q30 above 91.75% from mRNA libraries of leave samples which were mentioned above. Raw data has been uploaded to the Sequence Read Archive as a BioProject (PRJNA875963). These clean reads were mapped into the *Camellia sinensis* genome (GCF_004153795.1), of which the mapping rate of each sample in this study was ranging from 83.60 to 90.29%, indicating that the reference genome will meet the research needs, and a total of 13,954 expressed unigenes (Both Read count and FKPM ≥ 1) were obtained. Genes with differential expression patterns between sensitive and tolerant cultivars (cutoff fold change ≥ 2 and *p*-value ≤ 0.05) were defined, and a total of 1,992 differentially expressed genes (DEGs) during the drought stress treatment, including 496 and 1,768 DEGs were detected in XL1 and HD2, respectively (Figure 2). Meanwhile, a total of 1,526 DEGs at different stages during the artificial simulation drought stress treatment among two different cultivars, including 121, 331, 268, and 1,161 DEGs after 0, 4, 8, and 12 days of drought stress treatment, respectively.

### Functional annotation and classification of differentially expressed genes

**FIGURE 3:**
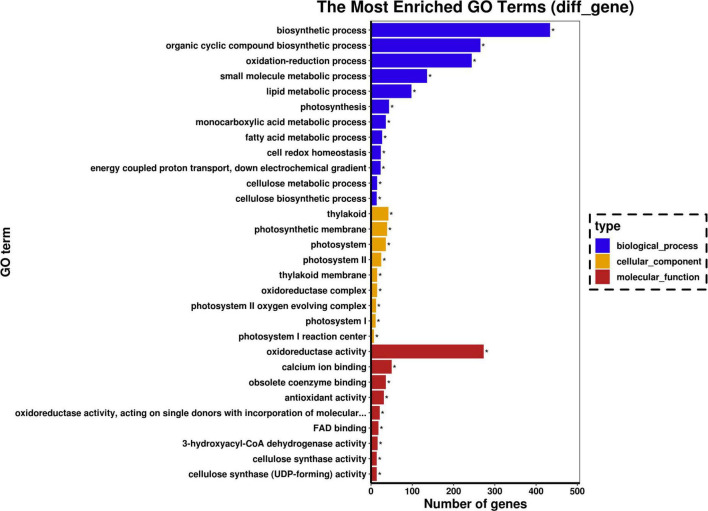
Gene ontology (GO) analysis for significant DEGs. Graphical representation of numbers of DEGs enriched in biological process, cellular component, and molecular function GO-terms. The symbol “*” represents the significantly enriched GO terms.

According to GO functional enrichment analysis, more than 33 GO biological process terms were significantly enriched, including biosynthetic, organic cyclic compound biosynthetic, oxidation-reduction small molecule metabolic, lipid metabolic process, and photosynthesis (Figure 3). The cellular component categories of DEGs were significantly enriched for the thylakoid, photosynthetic membrane, photosystem, and photosystem II. Significantly enriched GO molecular terms included oxidoreductase activity, calcium ion binding, obsolete coenzyme binding, and antioxidant activity.

**FIGURE 4:**
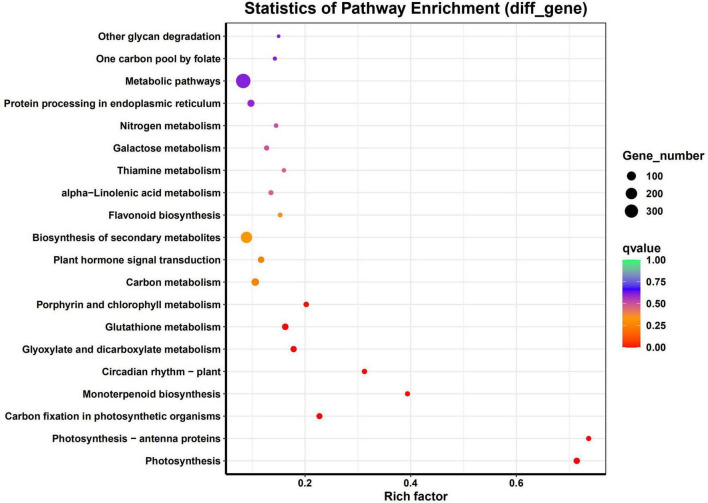
Pathway analysis for significant DEGs. Graphical representation of numbers of DEGs enriched in common KEGG pathway.

The DEGs were associated with various KEGG pathways involved in Photosynthesis, Photosynthesis - antenna proteins, Carbon fixation in photosynthetic organisms, monoterpenoid biosynthesis, circadian rhythm – plant, glyoxylate and dicarboxylate metabolism, glutathione metabolism, porphyrin and chlorophyll metabolism, carbon metabolism and plant hormone signal transduction (Figure 4).

### Differentially expressed genes associated with photosynthesis-related pathways

**FIGURE 5:**
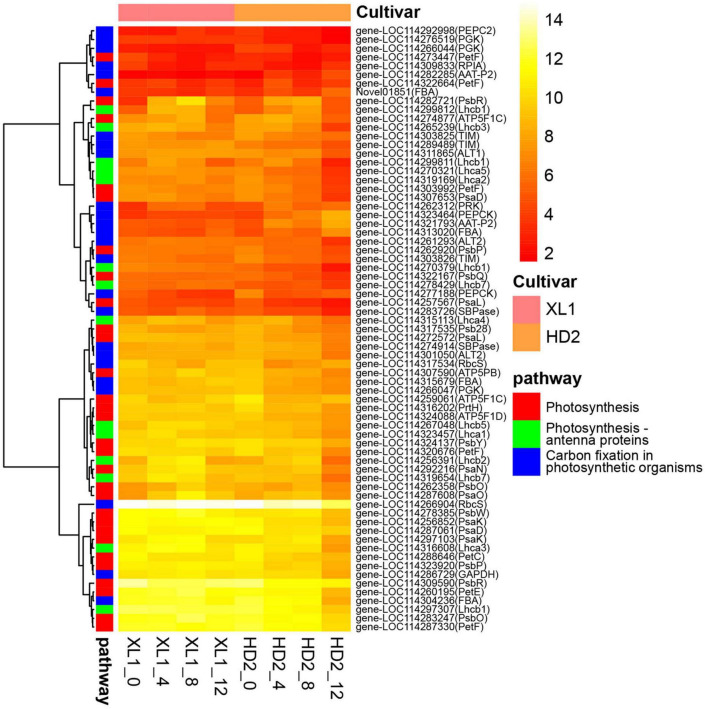
The heatmap of DEG involve in photosynthesis-related pathways. The heatmap was generated from the log_2_ (FPKM + 1) of three replicates for each sample, and the values of clustering distance were “correlation” and the clustering algorithms was “complete.”

Genes involving the photosynthesis-related pathways were divided into two groups (Figure 5). The majority of genes involved in photosynthesis and photosynthesis – antenna proteins were enriched in the first group. On the other hand, most of the genes involved in carbon fixation in the photosynthetic organisms pathway were enriched in the second group.

There were 9 DEGs encoded photosystem I reaction center subunit and 9 genes encoded photosystem II reaction center subunit, which down-regulated during the drought stress, meanwhile keeping higher expression levels in XL1 than that in HD2. There were 8 genes involved in photosynthetic electron transportation, including *PetC*, *PetE*, *PetF*, and *PetH*, whose expression patterns also showed that higher in XL1 than that in HD2 after drought stress.

There were 5 and 9 DEGs encoded proteins constructing LHCI and LHCII, respectively. The expression levels of *Lhca3*, *Lhca4*, *Lhca5*, *Lhcb1*, *Lhcb2*, *Lhcb3*, *Lhcb5*, and *Lhcb7* were up-regulated in XL1 at the first stages of drought stress, while those in HD2 were down-regulated.

Ribulose 1, 5-bisphosphate carboxylase/oxygenase (Rubisco), phosphoglycerate kinase (PGKs), glyceraldehyde 3-phosphate dehydrogenase (GADPHs), and fructose-bisphosphate aldolase (FBAs) played important roles in the CO_2_ fixation of the C3 plants. Two genes encoding Rubisco small chain were down-regulated in both XL1 and HD2 during drought stress with relatively higher expression levels in XL1 than that in HD2. Three *PGK* genes as well as the *GADPH* gene maintained a relatively stable expression pattern in XL1, whereas these genes were down-regulated in HD2 during drought stress. There were four genes encoding FBAs were identified, among these three genes were down-regulated in the first stages in XL1 and then up-regulated, whereas these genes were up-regulated in HD2.

Phosphoenolpyruvate carboxylase (PEPCs), phosphoenolpyruvate carboxykinase (PEPCKs), alanine transaminase (ALTs), and aspartate aminotransferase (AATs) played important role in the CO_2_ fixation in C4-dicarboxylic acid cycle. The *PEPC2* gene was up-regulated in XL1 and down-regulated in HD2 after drought stress. There were two genes encoding PEPCKs identified from DEGs, one of those was up-regulated in both XL1 and HD2, and the other was down-regulated in both XL1 and HD2. One *ALT1* gene and one of the *ALT2* genes were up-regulated in XL1 after drought stress, whereas these two genes were down-regulated in HD2. The rest ALT2 gene maintained a relatively stable expression pattern in XL1, while down-regulated in HD2. The *ATT* genes maintained a relatively stable expression pattern in XL1, while up-regulated in HD2.

### Differentially expressed genes associated with circadian clock system

**FIGURE 6:**
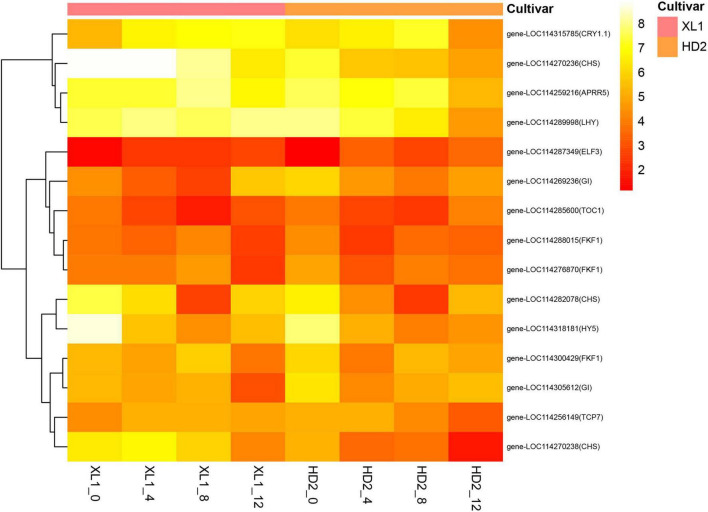
The heatmap of differentially expressed genes (DEGs) involve in the Circadian rhythm - plant pathway. The heatmap was generated from the log_2_ (FPKM + 1) of three replicates for each sample, and the values of clustering distance were “correlation” and the clustering algorithms was “complete.”

There were 15 DEGs enriched in the circadian rhythm – plant pathway (Figure 6), among these genes the *Cryptochrome* (*CRY*) and *Early Flowering 3* (*ELF3*) encoding proteins participated in the input pathway, while the *Late Elongated Hypocotyl* (*LHY*), *Timing of CAB expression 1* (*TOC1*), *pseudo-response regulator 5* (*PRR5*) and *GIGANTEA* (*GI*) encoding proteins that constructed core feedback loop in the oscillator.

The gene expression levels of *CRY1.1* were up-regulated after drought stress in both XL1 and HD2, while its downstream regulatory gene *ELF3* was down-regulated. The *LHY* gene expression was up-regulated in XL1 and down-regulated in HD2 after drought stress, respectively, while the expression levels of its repressor gene *TOC1* were down-regulated in both XL1 and HD2. The expression levels of *APRR5* and *GI* showed no significant differences at the first stages after drought stress in XL1, which showed significantly down-regulated in HD2. The Chalcone Synthase (CHS) acted as an output pathway regulator, there were three *CHS* genes differentially expressed during drought stress between XL1 and HD2, among these genes down-regulated after 4 days in XL1 and 8 days in HD2, respectively.

### Differentially expressed genes associated with phytohormone signal transduction

**FIGURE 7:**
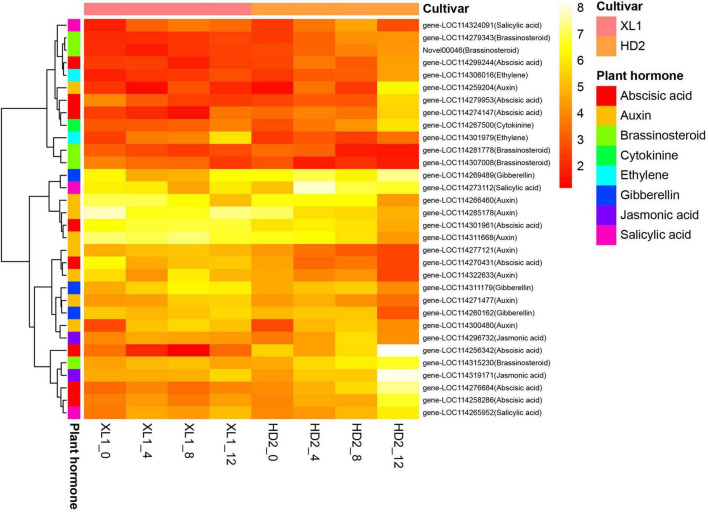
The heatmap of differentially expressed genes (DEGs) involve in the Plant hormone signal transduction pathway. The heatmap was generated from the log_2_ (FPKM + 1) of three replicates for each sample, and the values of clustering distance were “correlation” and the clustering algorithms was “complete.”

A total of 32 DEGs involved in several plant hormone signal transduction pathways were identified (Figure 7), including auxin, abscisic acid (ABA), brassinosteroid (BR), gibberellin (GA), salicylic acid (SA), ethylene (ET), jasmonic acid (JA) and cytokinin (CK), respectively. There were 8 genes involved in the auxin signaling pathway, among these genes 5 genes encoding auxin-responsive protein IAAs and 3 genes encoding auxin-responsive protein SAURs, respectively. The gene expression levels of three *IAAs* genes showed up-regulated at the first stages after drought stress and two *IAAs* gene were down-regulated in XL1, while the expression levels of all the five *IAAs* genes showed down-regulated in HD2. The ABA signaling pathway consisted of the abscisic acid receptor (PYR/PYL), protein phosphatase 2C (PP2C), sucrose non-fermenting 1-related protein kinase 2 (SnRK2), and their downstream regulatory ABA-responsive element binding factor (ABF). The expression level of *PYL6* gene which encodes an abscisic acid receptor was up-regulated in both cultivars after drought stress, while the expression levels in XL1 were higher than those in HD2 at each time point under drought stress, respectively. There were four genes encoding PP2Cs family protein, including *PP2C16*, *PP2C24*, *PP2C51*, and *PP2C75*, among these genes *PP2C24*, *PP2C51*, and *PP2C75* gene expressions were inhibited by drought stress in XL1, while *PP2C16*, *PP2C24*, and *PP2C51* gene expressions were induced by drought stress in HD2. The expression levels of *SnRK2.8* gene were significantly down-regulated at first in both XL1 and HD2, and then slightly recovered. The *bZIP8* and *ABI5* genes as downstream regulatory genes in the ABA signaling transduction pathway, their expression levels increased to a certain degree under drought stress, which indicated that the ABA signaling transduction pathway played a crucial role in the molecular response to drought stress. Another plant hormone signal transduction pathway participated in drought adaptation regulation involved with BR, the *BSK4* and *BSK8* genes as the BR signal receptor kinase encoding genes, their expression levels increased to a certain degree under drought stress, which indicated that the BR signal transduction pathway might be activated by drought stress. The *GSK2* and *BZR1* genes were up-regulated and down-regulated after drought stress in both cultivars, respectively, consequently, the downstream regulatory *CYCD3-1* gene was down-regulated after drought stress. In the SA pathway, the *NPR5* and *TGA2* genes were both up-regulated during drought stress in XL1 and HD2, while the downstream regulatory *PR-1* gene was down-regulated in XL1 and up-regulated in HD2 during drought stress, respectively. The key regulatory genes *DELLA* from GA pathway and *EIL* from ET pathway were both induced during drought stress in XL1 and HD2, indicating that these pathways might participate in downstream drought-responsive processes. The above results suggested that the plant hormone signal transduction pathway played important role in drought adaptation in *C. oleifera*.

### Drought stress responsive transcription factors

**FIGURE 8:**
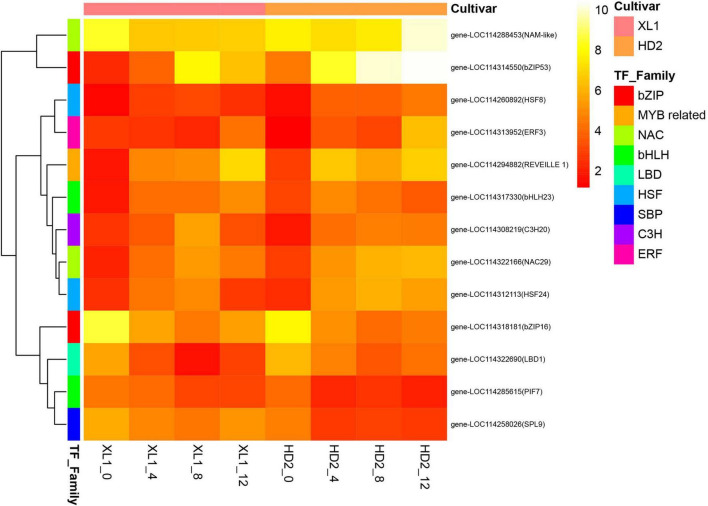
The heatmap of differentially expressed transcription factors (TFs) during the drought stress treatment. The heatmap was generated from the log2 (FPKM + 1) of three replicates for each sample, and the values of clustering distance were “correlation” and the clustering algorithms was “complete.”

In this study, a total of 150 transcription factors (TFs) were differentially expressed during the drought stress treatment. The most enriched TF families that responded to drought stress were bHLH, bZIP, C2H2, ERF, GATA, GRAS, HD-ZIP, MYB, MYB related, NAC, TCP, and WRKY. The MYB and MYB-related family was the largest group (12.00%), followed by the bHLH family (8.00%), WRKY family (8.00%), and NAC family (7.33%). There were 6 and 10 genes differentially expressed during the whole drought stress treatment in XL1 and HD2, respectively (Figure 8). The heatmap of differentially expressed TFs showed that there were 8 TFs significantly up-regulated including two from the HSF family (HSF8 and HSF24), and one from each of MYB related, bHLH, NAC, bZIP, C3H and ERF families, while the rest of differentially expressed TFs significantly down-regulated including TFs that mainly from the bZIP, NAC, LBD, bHLH, and SBP families.

### Weighted gene co-expression network analysis of differentially expressed genes

**FIGURE 9:**
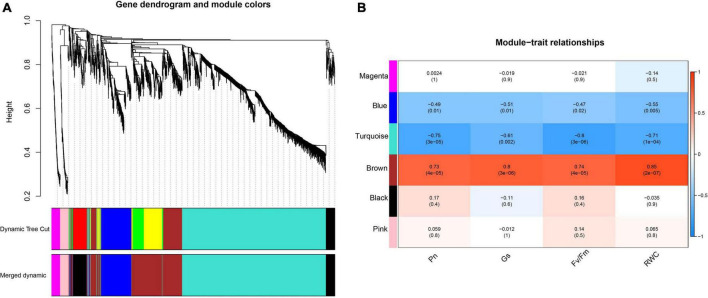
Network analysis dendrogram showing modules identified by WGCNA. **(A)** Hierarchical cluster tree showing 9 co-expression modules (including gray module) identified by WGCNAs, each branch in the tree represents an individual gene; **(B)** Correlation between module eigengenes and photosynthetic parameters, each cell contains the corresponding correlation and *p*-value.

The WGCNA result (Figure 9) indicated that DEGs could be grouped into 6 modules, which contained 1 327, 574, 285, 210, 77, and 77 in turquoise, brown, blue, black, magenta, and pink, respectively. Correlation analysis that conducted between physiological traits and module eigengenes (MEs) indicated that genes of brown module were significantly positive correlated to the photosynthetic physiological traits such as P_n_ (*r*^2^ = 0.73, *p* < 0.05), G_s_ (*r*^2^ = 0.80, *p* < 0.05) and Fv/Fm (*r*^2^ = 0.74, *p* < 0.05), as well as RWC (*r*^2^ = 0.85, *p* < 0.05). In contrast, genes clustered in turquoise module negatively correlated to P_n_ (*r*^2^ = −0.75, *p* < 0.05), G_s_ (*r*^2^ = −0.61, *p* < 0.05) and Fv/Fm (*r*^2^ = −0.80, *p* < 0.05), as well as RWC (*r*^2^ = −0.71, *p* < 0.05).

**FIGURE 10:**
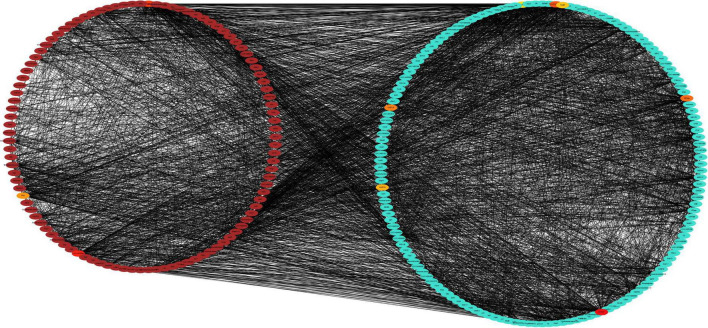
Protein-Protein Interaction network analysis of drought-responsive modules. PPI networks of the positively correlated brown module and the negatively correlated turquoise module were visualized using the Cytoscape software platform. The correlation networks of the top 10 genes and their directly regulatory genes from brown and turquoise modules were clustered into two circles. The color depth of 10 hub genes represents the strength of connectivity.

Brown and turquoise modules oppositely and positively correlated with photosynthetic physiological traits were selected to construct Protein-Protein Interaction (PPI) networks by enrichment analysis through the STRING database with the predicted proteins encoded by genes from the two modules (Figure 10). Functional enrichment in the PPI network showed that genes in brown and turquoise modules enrich in photosynthetic electron transport in photosystem I, negative regulation of response to salt stress, negative regulation of response to water deprivation, photosystem II assembly and gluconeogenesis Biological Process; chlorophyll-binding, rRNA binding, isomerase activity, calcium ion binding and oxidoreductase activity Molecular Function; photosystem II oxygen-evolving complex, photosystem II, photosystem and chloroplast thylakoid lumen Cellular Component. The KEGG pathways enriched with genes in brown and turquoise modules contained photosynthesis – antenna proteins, photosynthesis, thiamine metabolism, glyoxylate and dicarboxylate metabolism, and porphyrin and chlorophyll metabolism. These results suggest that photosynthesis-related genes were significantly affected during the drought stress, their differences between drought-tolerant and sensitive cultivars might play a crucial role in regulating drought adaptation in *C. oleifera*.

**FIGURE 11:**
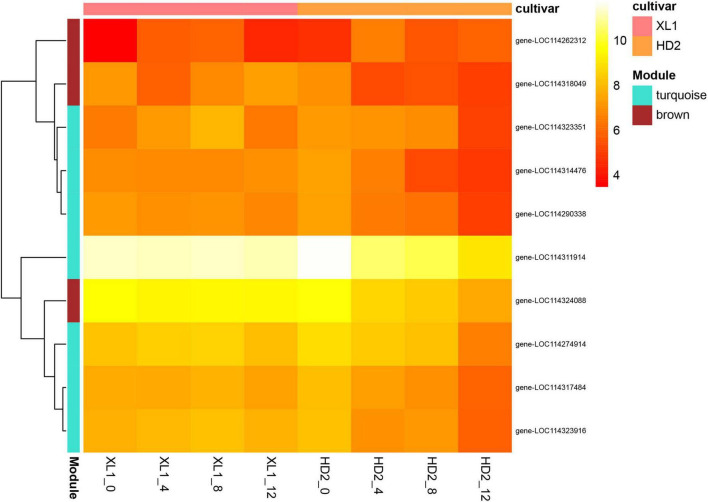
Expression levels of top ten hub genes of the PPI network. Heatmap showing the expression profiles of the top ten hub genes, the color depth of each cell represents the gene expression level by log_2_(FPKM + 1), and the values of clustering distance were “correlation” and the clustering algorithms was “complete.”

To screen for candidate hub genes, the top ten connected genes in the selected modules were ranked by Maximal Clique Centrality (MCC) method with Cytoscape (Figure 11), resulting in 3 genes from the brown module and 7 genes from the turquoise module were screened out, respectively. In the related brown module, the hub genes encoded Thylakoid lumenal 16.5 kDa protein, F-type h + -transporting ATPase subunit delta, and Phosphoribulokinase. In the related turquoise module, the hub genes encoded Thioredoxin-dependent peroxiredoxin, Thylakoid lumen 18.3 kDa family protein, Sedoheptulose-1,7-bisphosphatase, Curvature Thylakoid 1c, Thylakoid membrane phosphoprotein 14 kDa, Photosynthetic ndh subunit of subcomplex b 5 and an Uncharacterized protein. This indicates that these genes may play a crucial role in the regulatory network of drought stress adaptation.

### Identification of miRNAs in response to drought stress in *Camellia oleifera*

High-throughput Illumina sequencing yielded a total of 52,492,335 unique reads in the miRNA libraries, and sequences with lengths ranging from 18 to 35 nt were selected for the following analysis. Mapped small RNA tags were used to look for known miRNAs against miRase and 3,051 known miRNAs from 64 miRNA families were identified. In addition, *de novo* predictions have been performed that identified another 241 miRNA candidates.

To explore the differential expression of miRNAs related to drought stress in XL1 and HD2, 83 expressed miRNAs (readcount ≥ 1 in each sample), including 55 known miRNAs and 28 novel miRNAs, were conducted differential expression analysis by DESeq2 package. Comparisons with control groups revealed 7 and 5 differential expressed miRNAs (DEMs) in XL1 and HD2, respectively (cutoff fold change ≥ 1 and *p*-value ≤ 0.05). Among these DEMs, 5 were identified as known miRNA mainly from miR398 and miR408 families, while 2 were predicted as novel miRNA candidates. Known and novel miRNAs with differential expression patterns between sensitive and tolerant cultivars were defined, a total of 14 miRNAs during the artificial simulation of drought stress treatment, including 0, 6, 6, and 5 miRNAs after 0, 4, 8, 12 days of drought stress treatment, respectively, were identified as DEMs between two cultivars. Among these DEMs, 11 were identified as known miRNA from miR159, miR166, miR395, miR398, miR408, miR482, miR4995, and miR5368 families, while 4 were predicted as novel miRNAs.

### Target prediction and construction of a regulatory and interaction network

Differentially expressed miRNAs target gene prediction and GO annotation analysis was performed to characterize the regulatory roles of miRNAs in response to drought stress, and a total of 78 target DEGs were found. For these target DEGs, among the GO terms identified in the biological process, eight significantly enriched categories were “generation of precursor metabolites and energy,” “macromolecule localization,” “cellular component organization,” “cellular component organization or biogenesis,” “organic substance transport,” “cellular protein localization,” “cellular macromolecule localization” and “response to stress.”

**FIGURE 12:**
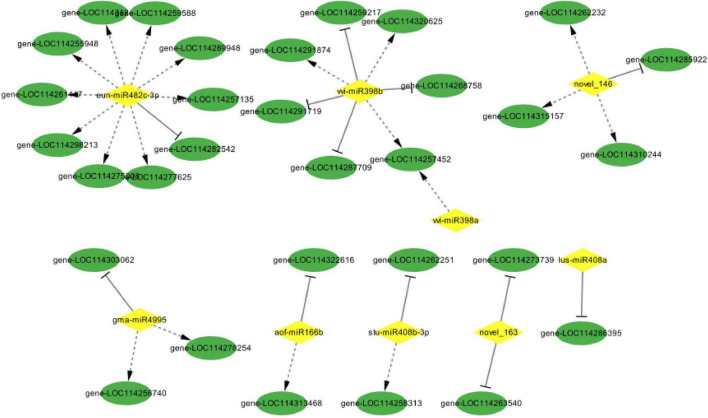
miRNA-mRNA regulatory network involved in the response to drought stress. The network of differentially expressed miRNAs and the predicted target genes was shown, and the negative and positive correlations between DEMs and their target were reflected by the solid line with a stop symbol and dotted line with an arrow, respectively.

To establish the regulatory network of miRNA-mRNA involved in the response to drought stress, the predicted target DEGs of DEMs were analyzed (Figure 12). The correlation analysis of expression levels was conducted between DEMs and the predicted target DEGs, 33 miRNA-mRNA pairs were screened out, involving 9 DEMs and 32 DEGs. Among these pairs, 12 pairs showed antagonistic regulatory patterns due to their significantly negative correlation-ship with expression levels (*p* < 0.05) (Table 2).

**TABLE 2:** miRNA-mRNA regulatory network response to drought stress in *Camellia oleifera*.

miRNA	Target	Description	R^2^	*p*-value
novel_146	LOC114285922	Acyl-homoserine-lactone synthase	−0.51	0.011
vvi-miR398b	LOC114259217	DOWNY MILDEW RESISTANCE 6-like	−0.50	0.013
stu-miR408b-3p	LOC114262251	ENHANCED DISEASE RESISTANCE 2	−0.50	0.014
vvi-miR398b	LOC114291719	Phosphoinositide phosphatase SAC1-like	−0.47	0.022
aof-miR166b	LOC114322616	Helicase sen1-like	−0.46	0.025
gma-miR4995	LOC114303062	Peroxisomal (S)-2-hydroxy-acid oxidase GLO4-like	−0.46	0.025
novel_163	LOC114273739	pantoate-beta-alanine ligase	−0.45	0.027
lus-miR408a	LOC114286395	vacuolar protein sorting-associated protein 53 A-like	−0.44	0.032
novel_163	LOC114263540	NADPH–cytochrome P450 reductase-like	−0.43	0.035
vvi-miR398b	LOC114268758	mitochondrial import receptor subunit TOM40-1-like	−0.41	0.045
eun-miR482c-3p	LOC114282542	Receptor-like protein kinase	−0.41	0.046
vvi-miR398b	LOC114287709	Bifunctional aspartate aminotransferase and glutamate/aspartate-prephenate aminotransferase-like	−0.41	0.049

### Verification of differentially expressed genes and differentially expressed miRNAs analysis results by quantitative real-time PCR

**FIGURE 13:**
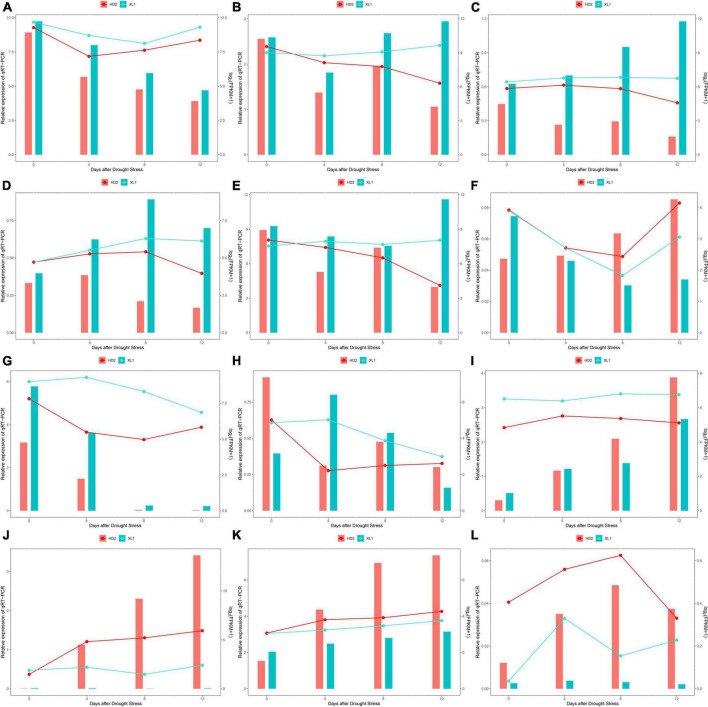
The comparison of the expression levels of 6 DEGs, 3 DEMs and their target genes identified response to drought stress among mRNA-Seq/miRNA-seq (line) and qRT-PCR analysis (bar). **(A)** *Rbcs* gene expression levels during the drought stress by qRT-PCR and mRNA-seq quantifications. **(B)** *FBA* gene expression levels during the drought stress by qRT-PCR and mRNA-seq quantifications. **(C)** *PYL6* gene expression levels during the drought stress by qRT-PCR and mRNA-seq quantifications. **(D)** *DELLA* gene expression levels during the drought stress by qRT-PCR and mRNA-seq quantifications. **(E)** *LHY* gene expression levels during the drought stress by qRT-PCR and mRNA-seq quantifications. **(F)** *TOC1* gene expression levels during the drought stress by qRT-PCR and mRNA-seq quantifications. **(G)** miR398 gene expression levels during the drought stress by qRT-PCR and mRNA-seq quantifications. **(H)** miR408-3p gene expression levels during the drought stress by qRT-PCR and mRNA-seq quantifications. **(I)** miR166 gene expression levels during the drought stress by qRT-PCR and mRNA-seq quantifications. **(J)** DMR6 gene expression levels during the drought stress by qRT-PCR and mRNA-seq quantifications. **(K)** EDR2 gene expression levels during the drought stress by qRT-PCR and mRNA-seq quantifications. **(L)** LOC114322616 gene expression levels during the drought stress by qRT-PCR and mRNA-seq quantifications.

To validate the reliability of the miRNA-seq and mRNA-seq data, 8 DEGs and 4DEMs that showed significant differences in expression were selected: *RbcS* and *FBA* from carbon fixation in photosynthetic organisms pathway (Figures 13A,B), *PLY6* and *DELLA* from plant hormone signal transduction pathway (Figures 13C,D), *LHY* and *TOC1* circadian rhythm - plant pathway (Figures 13E,F), miR398, miR408-3p, miR166 (Figures 13G–I), and their predicted miRNA candidates (Figures 13J–L). We analyzed the variance of expression of DEGs and DEMs in samples during the whole drought stress treatment using RT-PCR. The mRNA-seq and RT-PCR data were very closely correlated, and there was high consistency in the up- and down-regulated expression of DEGs and DEMs.

## Discussion

### Photosynthetic physiology traits response to drought stress in *Camellia oleifera*

Photosynthesis-related pathways consisted of photosynthetic pigments and photosystems, electron transport chain, and carbon dioxide reduction pathways. Damages to any of these components reduce the overall synthetic capacity of plants (Siddique et al., 2016). Plants perceive drought through chemical and hydraulic signaling and trigger chemical signaling from the root toward the shoot to initiate necessary molecular responses (Mukarram et al., 2021). These responses induce morpho-physiological changes, including stomatal behavior, and prepare the plant for the upcoming drought (Janiak et al., 2016). In general, dynamic regulation of the stomatal aperture prevent excessive transpirational water loss and minimizes the drought impact (Lawson and Matthews, 2020). Stomatal conductance provides an easy, integrated measure of the physiological response of plants to drought stress (Gilbert and Medina, 2016). The stomatal closure is triggered when plants suffered water deficiency, consequently, plants prioritize survival over growth and development. In this study, the decrease of G_s_ soon after drought stress in XL1 and HD2 reflected that both cultivars initiated the active closure mechanism to prevent water loss for further damage. Plants sacrifice photosynthetic capacities for survival when they undergo severe drought stress in the way of active stomatal closure that leads to less uptake CO_2_ abilities. In the comparison of the two cultivars, the G_s_ remain at higher levels in XL1 than that in HD2, while the P_n_ and T_r_ similarly reduced within the deepening drought stress with consistently higher levels in XL1 than that in HD2, indicating that XL1 remains higher photosynthetic capacities than HD2. Dark-adapted maximum photochemical efficiency of PSII (Fv/Fm) is a useful indicator of past stress in leaves and acclimation. Water deficiency limits the efficacy of the photosynthetic apparatus, causes substantial damage to the thylakoid membrane, and reduces the Chl contents of leaves, thus affecting the photoinhibition/photochemical intensity, resulting in reduced Fv/Fm (Muhammad et al., 2020). Fv/Fm depression is sustained and may decrease photosynthetic rates and thus also represent damage (Demmig-Adams et al., 2012). In this study, the Fv/Fm ratio significantly decreased in both cultivars after drought stress with a relatively higher level in comparison to XL1 and HD2, which indicated that HD2 suffered more severe damages than XL1 within the photosystem and electron transport chain.

### Differentially expressed transcription factors under drought stress in *Camellia oleifera*

Transcription factors (TFs), as the binding to the cis-regulatory elements in promotors to mediate the expression of massive genes, have fundamental importance in critical aspects of plant function in ensuring that plant growth and development match fluctuations in environmental conditions (Hrmova and Hussain, 2021). Notably, fully sequenced genomes of several plants showed that around 10% of all identified genes encode TFs (Baillo et al., 2019). In this study, a large number of DEGs encoding members of TF families including bHLH, bZIP, MYB, NAC, and WRKY, were identified according to their significantly responsive expression changes during drought stress. MYB TFs as one of the largest protein families in plants have been found to participate in diverse processes such as plant development and responses to biotic and abiotic stresses (Guo et al., 2022). In this study, we have found a drought-responsive differentially expressed gene encoding Reveille1 (RVE1), a Myb-like, clock-regulated TF that is homologous to the central clock genes *Circadian Clock Associated 1* (*CCA1*) and *Late Elongated Hypocotyl* (*LHY*). A previous study revealed that *RVE1* acts as a clock output instead of a central oscillator, due to the disordered expression of *RVE1* does not affect circadian rhythmicity (Rawat et al., 2009). The Phytochrome Interacting Factor (PIF) is a small subset of basic helix-loop-helix TFs that function as a cellular signaling hub, integrating internal and external stimuli to coordinate the regulation of the transcriptional network that drives multiple morphogenic responses (Leivar and Quail, 2011). The *PIF4*, *PIF5*, and *PIF7* have been shown that act as clock output controlled by circadian clock genes that manipulated plant growth and development (Cheng et al., 2021; Favero et al., 2021). It is reported that *PIF7* negatively regulates *DREB1* expression under circadian control in *Arabidopsis*, consequently repressed *DREB1* expression may be important for avoiding plant growth retardation under unstressed conditions. In this study, a *PIF7* gene significantly decreased in HD2 under drought stress, while it has no significant differences in XL1. These indicate that the circadian clock system might involve in the differences in drought stress response.

### miRNA-mRNA network under drought stress in *Camellia oleifera*

Plants have developed the function of using miRNA to regulate gene transcription in response to environmental factors and stress conditions such as temperature, light, water, and nutrient deficiency (Kryovrysanaki et al., 2022). Among the screened differentially expressed miRNAs in this study, miR398 played an important role in the regulatory network that responds to drought stress in *C. oleifera*. Several studies have been made for the identification of drought-responsive miRNAs, and members from the miR398 family have been proven to be one of the most important stress-responsive miRNAs, which play important roles in oxidative stress, drought stress, salt stress, ABA signal, copper and phosphate deficiency, as well as biotic stress (Bakhshi et al., 2014). Quantitative expression analysis of miRNAs during drought stress in *Camellia sinensis* carried out with the most famous oolong tea cultivar ‘Tieguanyin’ by former researchers revealed that members from miR166, miR398, and miR408 families were possibly associated with the *C. sinensis* drought stress (Guo et al., 2017), which provided evidence that supported the credibility of our results. Interestingly, the expression levels of miR166 and miR398 were down-regulated and up-regulated during the different phases of drought stress, respectively, which were exactly the opposite of those in *C. oleifera* from our results. Commonly, the network of miRNAs could be the same in different plant species, or it may differ between species (Akdogan et al., 2016). The consistent results were obtained from research conducted in *Echinacea purpurea* L., which showed that miR398 expression was reduced in comparison with the control in different field capacities of water (Moradi and Khalili, 2019). Furthermore, a previous study illustrated that miR398 targets Cu/Zn-superoxide dismutase (CSD1 and CSD2) which were found to be significantly induced in the leaf of wheat cultivars to provide drought tolerance (Akdogan et al., 2016). In this study, the expression level of miR398b negatively correlated to those of gene-LOC114259217, gene-LOC114291719, gene-LOC114268758, and gene-LOC114287709, which encoding a DOWNY MILDEW RESISTANCE 6-like protein, a phosphoinositide phosphatase SAC1-like protein, a mitochondrial import receptor subunit TOM40-1-like protein and a glutamate/aspartate-prephenate aminotransferase-like protein. The significant decreases of miR398b were observed and consequently led to increasing the expression levels of the predicted target genes mentioned above. Besides, the similar expression between other predicted target genes and miRNAs indicated the inconsistent regulation of miRNA, these might be mostly due to the expression of unknown TFs and activation of other metabolic networks in the drought stress resistance network (Moradi and Khalili, 2019). In this study, these unexpected regulatory networks are mainly involved in gene-LOC114257452, gene-LOC114320625, and gene-LOC114291874, which encode THO complex subunit 4A, Chloroplast Unusual Positioning 1 protein, 60S ribosomal protein L44-like.

miR408 is a multistress-responsive miRNA that observed to be differentially regulated in plants such as *Arabidopsis* (Song et al., 2017), *Triticum* (Feng et al., 2013), *Oryza* (Balyan et al., 2022), *Chickpea* (Hajyzadeh et al., 2015), and *Vigna unguiculata* (Mishra et al., 2021). Previous studies showed that overexpression of miR408, by down-regulation of the direct target genes, obviously promotes photosynthetic activity, biomass, and seed yield (Song et al., 2017; Pan et al., 2018), as well as tolerance to drought stress (Hang et al., 2021). However, another study of overexpression mutant revealed that higher miR408 expression led to enhanced sensitivity to drought stress (Hajyzadeh et al., 2015; Ma et al., 2015). This opposite role of miR408 in plant drought-responsive regulatory networks might indicate a species-specific drought-responsive characteristic for miR408 (Mutum et al., 2013). The miR408b-3p identified in our study showed that it was down-regulated after drought stress, and the abundance in XL1 was consistently higher than that in HD2. The miR408-3p induced its predicted target gene which encodes protein Enhanced Disease Resistance 2 by negatively regulating of decreasing itself, might further improve drought tolerance *via* crosstalk between different stress-responsive pathways.

## Data availability statement

The datasets presented in this study can be found in online repositories. The name of the repository and accession number can be found below: NCBI Sequence Read Archive; PRJNA875963.

## Author contributions

ZH and CL: software and writing—review and editing. ZZ: validation and data curation. RW and ZH: investigation. ZH: writing—original draft preparation. YC: supervision, project administration, and funding acquisition. All authors read and agreed to the published version of the manuscript.
